# Technical and locomotor demands in elite soccer: manipulating area per player during small-sided games to replicate official match demands

**DOI:** 10.5114/biolsport.2023.118338

**Published:** 2022-09-15

**Authors:** Andrea Riboli, Fabio Esposito, Giuseppe Coratella

**Affiliations:** 1 Department of Biomedical Sciences for Health, Università degli Studi di Milano, Milan, Italy

**Keywords:** Team sports, Football, Performance, Match analysis, Technical analysis

## Abstract

The present study aimed to investigate the area per player (ApP) to replicate the technical and locomotor match demands using small-sided games (SSGs) in male soccer players (*n* = 20) competing in major European and UEFA competitions. The relative number of each individual technical activity per minute (number · min^−1^; technical demands) was counted and the relative (m · min^−1^) total (TD), high-speed running (HSRD), very high-speed running (VHSRD), sprint and acceleration+deceleration (Acc+Dec) distances were collected during different SSG formats (*n* = 24; 4 vs 4 to 10 vs 10 with an ApP from 60 to 341 m^2^ · player^−1^) and official matches (*n* = 28). Data were collected during two full seasons. A linear mixed model analysis was used to calculate the individual relationship between technical/locomotor demands and the ApP during SSGs; the correlation coefficient was also calculated. With the exception of an inverse *moderate* (*r* = -0.457) correlation for Acc+Dec, each locomotor metric (TD, HSRD, VHSRD and sprint) showed a positive *large* to *very large* (*r* = 0.560 to 0.710) correlation with ApP (*P* < 0.001). The technical demands showed an inverse moderate correlation (*r* = -0.529) with ApP. Additionally, inverse *moderate* to *large* correlations (*r* = -0.397 to -0.600; *P* < 0.05) between the technical demands and the locomotor demands (TD, HSR, VHSR and sprint) were found. Lastly, an ApP of ~243 m^2^ · player was found to replicate the official match technical demand and it was quite similar to the ApP required to replicate HSRD, VHSRD and sprint. These findings may help practitioners to replicate, overload and underload both technical and locomotor demands using a specific ApP during SSGs in elite soccer.

## INTRODUCTION

In modern soccer, the training prescriptions follow a game-based approach in which ball-drill prescriptions are focused on the game as a whole [1]. In this approach, key learning occurs from the game itself and game-related activities [2]. The aim is not only to teach the skills required to play a game, but also to allow one to develop the ability to understand the game’s tactics and strategies within a highly specific environment [2]. In this regard, small-sided games (SSGs) should be highlighted due to their ability to integrate physical fitness, technical, tactical and conditional stimuli in contexts similar to a real game [3–5]. Usually, SSGs are utilized as soccer-specific ball-drills [4, 6] for maximizing technical, tactical and physical performance [7, 8]. The manipulation of pitch sizes, number of players, specific rules, etc., may affect technical, tactical and physical demands [3, 4, 6]. Increase in pitch size and reduction of the number of players were shown to increase the total distance and the distance covered at different speeds [9, 10]. Moreover, as the dimensions of the pitch increase in SSGs, the tactical principles such as penetration, defensive balance and defence unity become more frequent [11], and the periods in which teams retain possession of the ball become longer [12]. Conversely, when the pitch size is reduced and the number of players is increased, players perform more technical activities (e.g., stops, passes, shots, intercepts) and the prevalence of the locomotor demands tends to be characterized by accelerations and decelerations [4, 9].

During the training interventions using SSGs, manipulating the area per player (ApP, expressed as m^2^ × player) [4, 6, 13] enables practitioners to design the SSGs for different purposes [4, 13]. It was recently shown that ApP during SSGs was *very largely* correlated with the relative (m · min^−1^) total distance, high-speed running and sprint covered, while no correlation for acceleration/deceleration was found both in adult [4] and youth [5] elite soccer players. This was in line with previous findings suggesting that SSGs in a higher ApP resulted in stronger physical and physiological responses [10]. Additionally, a specific high ApP (i.e. ~300 m^2^ × player) was reported to induce internal/external load responses near to the individual maximal physiological capacities [14], to replicate official match metabolic and cardiovascular responses [15], to replicate official match sprint distances [4] and to simulate match tactical behaviours [13]. Moreover, an ApP similar to official match demands has been suggested to replicate the 4-min match-derived maximal intensities in elite soccer players [16]. Therefore, a large specific ApP was suggested to increase locomotor [4, 5], physiological [14] and tactical [3] demands up to the maximal official match demands [16] through a soccer-specific learning environment [5].

Although soccer requires technical, tactical and physical capacities, technical abilities such as stops, passes and shots are some of the main performance factors [17]. During official matches, several technical data (e.g. number of shots, passes, crosses) are usually collected to inform coaches and players about the individual and team technical performance [18]. Similarly, technical metrics have been collected to determine the individual activities with the ball during different formats of SSGs [6, 19, 20]. In this regard, the technical demands increased with small pitch sizes or with a lower number of players [21]. For these reasons, small pitch sizes are usually used to increase the amount of the individual technical activities during a soccer-specific training routine. Indeed, two reviews reported that ApP of ~91 m^2^ · player [4] or ~93 m^2^ · player [24] during SSGs is suitable for these purposes. However, these may lower the locomotor demands, leading to a possible mismatch between SSGs and match-play requirements, especially in the high-speed to sprint activities [22]. Consequently, the use of small ApP could affect the physiological [14], physical [4, 5] and tactical [13] responses, reducing the training specificity usually advocated as a key factor of SSGs.

An integrated approach considering technical, tactical and physical demands contextualized across the official match performance requirements could be useful to maximize the development of the physical performance using SSGs [4, 5, 9]. Unfortunately, none of the previous studies coupled the technical with the locomotor demands to suggest a specific ApP to replicate both the technical and the locomotor activities typically required during official matches. Therefore, the present study aimed to determine the minimal ApP that could be used to replicate both the official match technical (i.e. the combined number of stops, passes, shots, crosses, tackles, etc. per minute per player) and locomotor demands in elite soccer players.

## MATERIALS AND METHODS

### Participants

Twenty-five elite soccer players competing in major European and UEFA competitions were included in the present study (age: 26 ± 6 years; body mass: 80 ± 7 kg; body height: 1.85 ± 0.08). As an inclusion criterion, each player should have played a minimum of six official matches for at least 85 minutes. Goalkeepers were excluded from the data collection. The club’s medical staff certified the health status of each player. As an exclusion criterion, an injured player was excluded from data collection for at least one month after their return to full training with the team. The procedures were fully explained to the participants and the club staff. The participants gave their written consent. The Ethics Committee of the Università degli Studi di Milano (protocol #102/14) approved the study, which was performed in accordance with the principles of the Declaration of Helsinki (1975) for studies involving human subjects.

### Experimental design

The present investigation was carried out during the competition period across two consecutive seasons. To avoid possible variation in training status, only the data collected during the in-season period were collected; to avoid possible individual cardiorespiratory and metabolic variations, the individual fitness status was continuously monitored across the season by a high-qualify technical staff, as previously reported [23]. The magnitude of the individual changes in fitness status (i.e. either a significantly higher or lower fitness level) was calculated on individual bases as previously proposed [23] and used as an exclusion criterion.

The participants undertook their traditional weekly training routine (5 training sessions, 1 match day and 1 day off). Only data collected during the training routine with one match per week were utilized for the aims of the current study. To avoid any fatigue-induced variations in the technical and locomotor demands, only the SSGs completed on the match day +3 after a standardized warm-up were considered for the current investigation (match day +1 and +2 were a recovery low-intensity training session and a day off, respectively). All sessions were performed on grass pitches preserved by qualified operators and were conducted at the same time of day to limit the effects of circadian variation. A specialized and high-qualified physician recommended and monitored the diet regime of each player before and after every training session.

A total of 1332 individual observations across 24 different formats of SSGs were undertaken. SSGs ranged from 4 vs 4 to 10 vs 10 with an ApP from 60 m^2^ to 488 m^2^. A detailed description of the SSGs’ characteristics is reported in Table 1. The ApP was calculated excluding the goalkeepers in SSGs. Each SSG lasted on average 4 minutes. The SSGs were performed under the supervision and motivation of several coaches to maintain a high work rate. For the same reason, a ball was always available by prompt replacement when it went out-of-play [6]. In SSGs, the corners were replaced by a prompt ball-in-game from the goalkeeper. The SSGs were completed after a standardized 20-min warm-up under the guidance of club staff. A total of 28.2 ± 5.4 official match individual samples were monitored. The official match pitch size was 105 × 66 m, with a grass surface.

**TABLE 1:** Pitch size during small-sided games with goalkeepers.

N° Players	m	Width:length ratio	m^2^	m^2^ · player^-1^	N° measures	N° individual samples
**10 vs 10**	65 × 52	0.8	3380	169	4	80
	65 × 60	0.923	3900	195	6	120
	105 × 65	0.619	6825	341	3	60

**9 vs 9**	40 × 36	0.9	1440	80	4	72
	52 × 40	0.769	2080	116	5	90
	65 × 45	0.962	2925	163	3	54
	65 × 52	0.8	3380	188	4	72

**8 vs 8**	35 × 30	0.857	1050	66	6	96
	65 × 40	0.615	2600	163	3	48
	65 × 45	0.692	2925	183	4	64
	65 × 60	0.923	3900	244	5	80
	70 × 65	0.929	4550	284	3	48

**6 vs 6**	40 × 36	0.9	1440	120	4	48
	52 × 36	0.692	1872	156	4	48

**5 vs 5**	30 × 20	0.667	600	60	5	50
	32 × 24	0.75	768	77	3	30
	30 × 30	1	900	90	5	50
	40 × 32	0.8	1280	128	4	40
	40 × 35	0.875	1400	140	5	50
	40 × 40	1	1600	160	3	30
	45 × 40	0.889	1800	180	3	30

**4 vs 4**	40 × 32	0.8	1280	160	6	48
	40 × 40	1	1600	200	3	24

The small-sided games with goalkeepers are split for the number of players and pitch size (length × width). **The width:length ratio**, the total pitch area (m^2^) and area per player (m^2^ · player ^−1^) have been calculated. **The total number of measures for each SSG format and the number of the individual samples are also reported.**

### Procedures

Video footage was recorded with high-definition dome cameras and high-resolution digital cameras (HDR-CX405, SONY Corporation, Minato, Tokyo, Japan). Each technical activity was counted by a specialized and high-qualified coaching staff with notational analysis during both training and match videos. To ensure the maximal accuracy in data collection, notational analysis was performed by three different UEFA B coaches (operators). A brief description for each technical event is reported in Table 2. The total number of technical activities was calculated as the combined number of technical activities and normalized by time (i.e. the number of technical activities per minute). The combined number of technical activities per minute was calculated within each SSG or match and calculated as technical activities per minute per player. Thereafter, we averaged the individual notational analysis to determine the corrected number of technical activities per minute per player.

**TABLE 2:** A brief description for each technical event is reported.

Technical events	Description
Total Activities	Number of technical activities that lead to any offensive or defensive actions carried out by the team in possession or non-possession of the ball. The total number of the technical activities was calculated as the combined number of the following activities and normalized by time (i.e. the number of technical activities per minute).

Shots	An attempt to score a goal, made with any (legal) part of the body either on or off target
Total passes	Number of an intentionally played ball from one player to another. All long, short, through passes and assists are considered.
Crosses	Number of long foot passes performed by a player from an offensive zone (last about 40 m of pitch between the short side of the penalty Area and the lateral side of the field) and direct to the penalty area.
Dribbling	Number of situations where a player tries to overcome another player with the ball possession.
Duels	Number of 1vs1 situations (with or without the ball) during with a player got in touch with the ball
Interceptions	Number of ball recoveries from an opponent’s ball possession by interception (interruption of an opponent ball transmission)
Recoveries	Number of ball recoveries from an opponent’s ball possession
Others	Any other voluntary ball touch during both possession or non-possession phases

A 10 Hz global positioning system unit (K-sport, Montelabbate, Italy) was used to collect data during training [4]. Each device was turned on at least 15 minutes before each session to allow for acquisition of the satellite signal [4]. The minimum acceptable number of available satellite signals was 8 (range 8–11), as previously suggested [24–26]. To reduce the inter-unit differences, each player wore the same unit for every training session over the whole investigation [4]. The locomotor activities during the official matches were collected using a computerized semi-automated video-based multi-camera image system (Stats Perform, Chicago, Illinois, USA) and processed by dedicated software [4]. The systems have previously been shown to provide valid and reliable measurements of the match activity in soccer [4, 27]. The GPS and the video-based multicamera image system were previoulsy detemined as interchangeable [4]. As previously reported [4], the magnitude of the bias between the GPS and the video-based multi-camera image system was *trivial* for each locomotor metric (about -3.0 to -3.9%, ES: -0.12 to -0.19).

During both training sessions and matches, total distance (TD), high-speed running distance (HSRD, 15 to 19.9 km × h^−1^), very-high speed running distance (VHSRD, 20 to 24 km × h^−1^), sprint distance (> 24 km × h^−1^) and acceleration+deceleration distance (Acc+Dec, > 3 m × s^−1^) were measured [4]. All data were normalized as meters covered in one minute (m · min^−1^) [4, 16].

To determine the ApP that replicates the normalized technical demands (technical activities × min^−1^), TD, HSRD, VHSRD, sprint and Acc+Dec (m × min^−1^) recorded during the official matches, we first recorded those variables during the official matches. Thereafter, we separately plotted each relationship between ApP and the normalized technical activities, TD, HSRD, VHSRD, sprint and Acc+Dec during SSGs. Then, the mean values recorded during the official matches were used to intersect each ApP/technical activity, TD, HSRD, VHSRD, sprint and Acc+Dec relationship recorded in SSGs to calculate the ApP that corresponded to the official match demands, as previously proposed [4, 5].

### Statistical analysis

SPSS (version 26, IBM Corp., USA) was used to perform the statistical analysis. To check the normal distribution of the sampling, the Shapiro-Wilk test was used.

The validity and reliability of the operators to detect the total number of the technical activities during both SSGs and official matches were determined as the between-operators typical error calculated as the coefficient of variation (CV%). The between-measures reliability was calculated using the interclass correlation coefficient (ICC) and interpreted as follows: < 0.50 poor reliability, 0.50 to 0.75 moderate reliability, > 0.75: good reliability [23].

A linear mixed model analysis was used to calculate the individual relationship between technical and locomotor (TD, HSRD, VHSRD, sprint and Acc+Dec) demands and the ApP during SSGs. The correlation coefficient between ApP and technical demands, TD, HSRD, VHSRD, sprint and acceleration/deceleration was calculated and interpreted as follows: *r* = 0.00–0.09 (*trivial*), 0.10–0.29 (*small*), 0.30–0.49 (*moderate*), 0.50–0.69 (*large*), 0.70–0.89 (*very large*), 0.90–0.99 (*nearly perfect*). Thereafter, a linear mixed model analysis was used to calculate the difference in the minimal ApP required to replicate technical demands, TD, HSRD, VHSRD, sprint and Acc+Dec. A post-hoc analysis (Holm-Sidak correction) was used to calculate the differences in the independent factors. Cohen’s *d* effect size with 95% confidence intervals (CI) was used to describe the magnitude of the pairwise differences and interpreted as follows: < 0.20 (*trivial*), 0.20–0.59 (*small*), 0.60–1.19 (*moderate*), 1.20–1.99 (*large*), ≥ 2.00 (*very large*). Statistical significance was set at α < 0.05. Unless otherwise stated, all values are presented as mean ± standard deviation (SD).

## RESULTS

The reliability level of the operators to detect the total number of technical activities using video footage showed a ~2.1(0.4)% CV and *good* ICC [~0.891(0.032)].

**FIG. 1:**
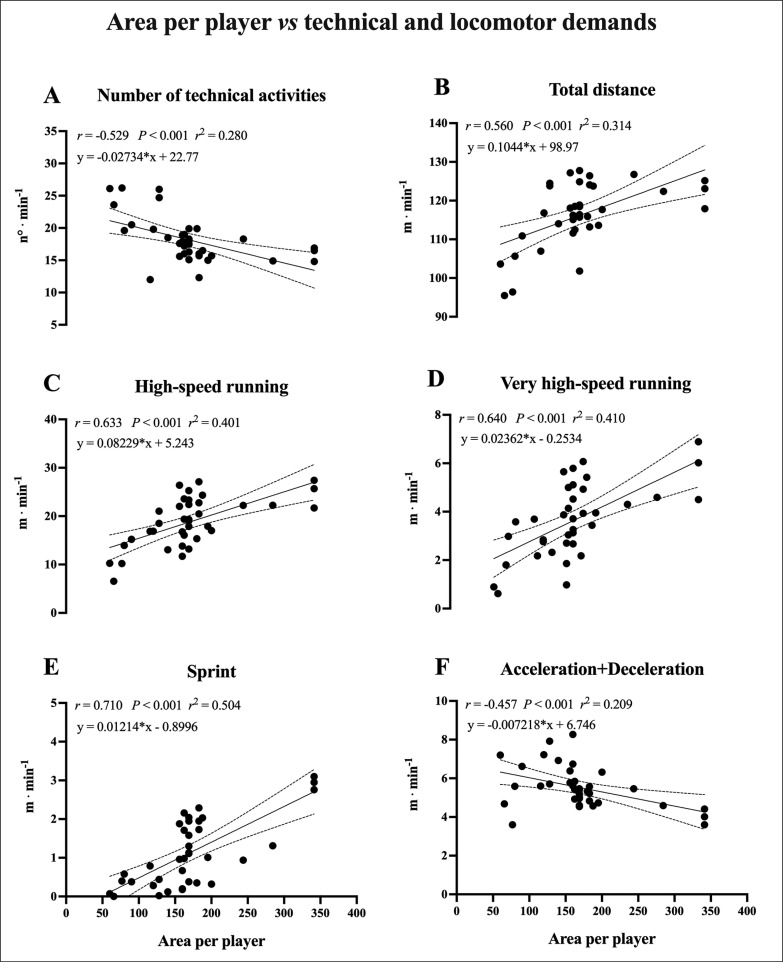
The relationship between area per player (m^2^ · player) and relative technical (technical activities · min^−1^) or locomotor (m · min^−1^) demands during small-sided games. The linear regression analysis with 95% confidence intervals and the correlation between the area per player and the relative technical or locomotor demands are reported for number of technical activities (Panel A), total distance (Panel B), high-speed running distance (Panel C), very high-speed running distance (Panel D), sprint distance (Panel E) and acceleration+deceleration distance (Panel F).

For technical demands, an inverse *moderate* correlation with ApP was found (Figure 1, panel A). For locomotor demands, the correlations with ApP were *large* for TD, HSRD and VHSRD (Figure 1, panel B, C and D), *very large* for sprint (Figure 1, panels E) and inversely *moderate* for Acc+Dec (Figure 1, Panel F).

**FIG. 2:**
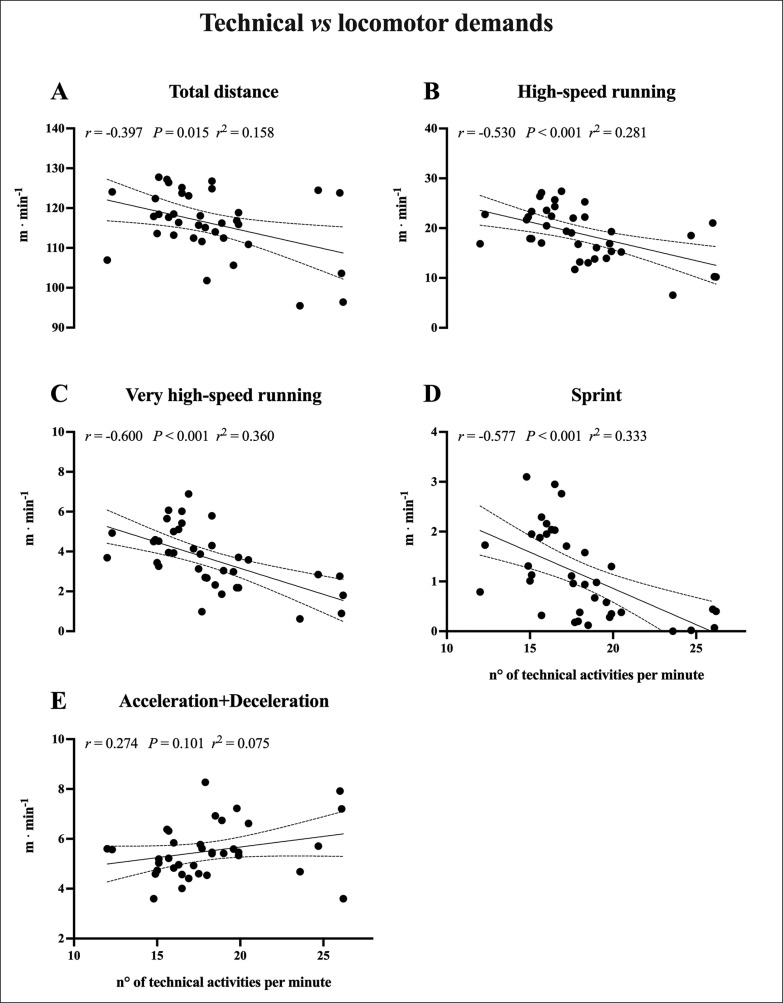
The relationship between relative locomotor (m · min^−1^) and relative technical (technical activities · min^−1^) demands during small-sided games. The linear regression analysis with 95% confidence intervals and the correlation between the relative locomotor and technical demands are reported for total distance (Panel A), high-speed running distance (Panel B), very high-speed running distance (Panel C), sprint (Panel D) and acceleration+deceleration distance (Panel E).

As presented in Figure 2, inverse *moderate* to *large* correlations (*P* < 0.05) between the number of technical activities per minute and locomotor demand were found for TD, HSRD, VHSRD and sprint.

**FIG. 3:**
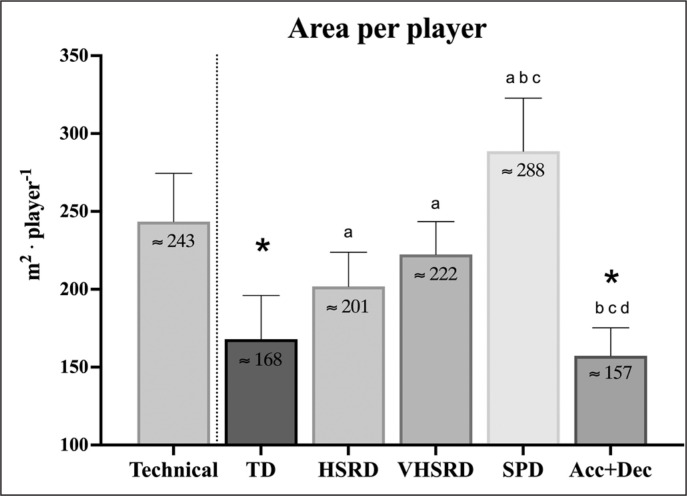
The minimal area per player (m^2^ · player) to replicate technical (technical activities · min^−1^) or locomotor (m · min^−1^) match demands using small-sided games. Data are reported as mean (SD). Technical: number of technical activities per minute per player; TD: total distance; HSRD: high-speed running distance; VHSRD: very high-speed running distance; SPD: sprint distance; Acc+Dec: acceleration+deceleration distance. ^*^P < 0.05 vs Technical. ^a^*P* < 0.05 vs TD; ^b^*P* < 0.05 vs HSR; ^c^*P* < 0.05 vs VHSR; ^d^*P* < 0.05 vs SPR.

As shown in Figure 3, ApP to replicate technical demands showed no differences compared to ApP for replicating HSRD, VHSRD and sprint; conversely, a higher (*P* < 0.001) ApP to replicate technical demands than TD (ES: 2.51; CI: 1.91 to 3.11) and Acc+Dec (ES: 3.36; CI: 2.66 to 4.06) was found. For locomotor demands, higher (*P* < 0.05) ApP for HSRD, VHSRD and sprint than TD (ES: 1.34 to 3.85) and Acc+Dec (ES: 2.22 to 4.80) were reported. Sprint required a higher (*P* < 0.001) ApP than each other locomotor metric (ES: 2.39 to 4.80).

## DISCUSSION

The present study investigated for the first time the optimal ApP in SSGs to reproduce both the technical and the locomotor match demands in elite soccer players. The number of technical activities was inversely correlated with ApP. As concerns the locomotor demands, TD, HSRD, VHSRD and sprint increased with increments in the ApP, while Acc+Dec decreased when incrementing the ApP. The minimal ApP to replicate the technical demands was ~243 m^2^ · player. Interestingly, the ApP to reproduce the same number of technical demands recorded during official matches was similar to the ApP for replicating HSRD (~201 m^2^ · player), VHSRD (~222 m^2^ · player) and sprint (~288 m^2^ · player). These findings may help coaches and sport scientists to manipulate ApP during SSGs to replicate both technical and locomotor official match demands.

The present results demonstrated that the increments in ApP reduced the number of technical activities per minute, affecting the technical intensity. Moreover, these findings showed an inverse relationship between the technical demands and each locomotor metric. It was previously reported that ApP manipulation is useful to modify the technical learning environment [28–30]. Indeed, SSGs put physical, technical and tactical skills into play to cooperate with team members competing with the opponent towards offensive and defensive phases [3, 31, 32]. As such, the ApP manipulation should help performance staff to train skills within a highly specific soccer environment [4, 5]. A lower number of blocks, headers, interceptions, passes and receives but more dribbles, shots and tackles were reported using an ApP of ~125 m^2^ · player than ~166 m^2^ · player [30]. Similarly, a higher number of tackles and shots with no differences for passes, receives, turns, dribbles, headers and interceptions was reported using an ApP ~75 than ~150 or ~250 m^2^ · player in English Championship players [29]. In the real-life training routine, small pitch sizes are usually prescribed to increase the number of duels as well as to complicate the technical challenges [33]. The use of SSGs with a reduced pitch size was reported previously to effectively train players in the technical aspects by allowing greater exposure in the time with the ball without excessive physical demands [28]. The current findings also showed an inverse relationship between the technical and the locomotor demands when manipulating the ApP (i.e. the smaller ApP, the higher the technical demands and the lower the TD, HSRD, VHSRD and sprint). A highly contextualized training prescription based on the real-game model using a specific ApP may help to recreate soccer-specific tasks to improve technical, tactical and physical abilities and the decision-making process across soccer-specific locomotor outcomes [1, 2]. Interestingly, the current findings determined the ApP of ~243 m^2^ · player as the optimal pitch dimension to replicate the technical demands during official matches. Interestingly, such an ApP was quite close to the ApP required to replicate HSRD (~201 m^2^ · player), VHSRD (~222 m^2^ · player) and sprint (~288 m^2^ · player). Therefore, with the intention to increase the technical stimuli, lower ApP could be used. This would overload the number of individual involvements with the ball compared with the official matches. Indeed, previous studies suggested an ApP of ~91 m^2^ · player [4] or ~93 m^2^ · player [24] for technical purposes during SSGs. In contrast, when the purpose is recreating the match conditions, an ApP of ~250 m^2^ · player could be utilized to replicate both technical and locomotor official match demands. This would imply that the ApP during the SSGs should be manipulated depending on the aims of the session.

The present findings remark that a larger ApP is warranted for the high-speed demands [4, 5] as previously reported both in adult [4] and youth [5] elite soccer players. This mainly depends on the large space necessary to reach high speeds during SSGs, while a small pitch size is sufficient to reproduce Acc+Dec [4, 5]. It should be noted that the Acc+Dec do not change meaningfully across the different ApP [4, 5, 16], so a larger ApP can still be used for stimulating both high-speed demands and Acc+Dec. Therefore, to replicate the whole official match demand including the sprint distance, an ApP of ~288 m^2^ × player appears to be needed. In line, a minimal ApP of ~311 m^2^ × player or ~316 m^2^ × player was indicated previously for sprinting in Italian Serie A [9] and French League One [4] soccer players. A specific ApP ~340 m^2^ × player has been recently suggested to replicate the official match peak demands [16]. Additionally, playing SSGs in an ApP ~320 m^2^ × player was also reported to enhance the players’ tactical organization during attacking and defending actions [3] and to replicate the physiological match demands [14, 15]. Therefore, a larger ApP implies greater distance covered at very highspeed running and sprinting [4, 16], and influences the players’ perception of space, conditioning its occupation and use, as well as the distances between players and their interactions [12] to create scoring opportunities in offensive situations and to avoid the opponents’ advance in defensive situations [3, 10].

Arguably, the exposure to the sprint activity occurring at a large ApP might be possibly protective against hamstring injuries [34], and should therefore be considered in practice. In this view, a large ApP appears most comprehensive in terms of the technical, tactical and physical official match demands [4, 5], preparing the players for the demands of the competition [31].

There are some limitations of the current investigation. Firstly, we would highlight that for replication purposes an individualized approach is required due to the typical soccer-specific variability (e.g., the athletes’ characteristics, coaches’ style of play) [23, 35], possibly affecting the current results both for the number of technical activities and locomotor demands; also the sample size of the measurements and the SSGs formats (different ApP, number of players, pitch size, width per length ratio, etc.) may affect both technical and locomotor demands during replicational studies. Secondly, for physical demands, the internal load parameters (e.g., heart rate) and the rate of perceived exertion were not examined. However, some technological limitations (e.g., the use of portable thoracic bands, especially during official matches) or some contextual limitations (e.g., athletes buy-in to collect rate of perceived exertion after each drill) affected the use of internal load assessment tools in the present study, especially during official matches. However, we acknowledge that coupling external load with internal load metrics may warrant a better understanding of drill demand. Thirdly, we would like to remark that to determine technical intensity the combined number of stops, passes, shots, crosses, tackles, etc. per min per player was utilized; however, future research could further investigate the effect of ApP on each single technical metric. Fourthly, individualizing the speed thresholds using the physiological individual profile [36–38] and/or individual maximal sprint speed [39] may help to further improve the understanding of the locomotor and physiological demands imposed on each player during different drills. Lastly, future studies may further investigate peak match demands [40] as a reference for SSGs, as recently proposed for the locomotor demands [16].

## CONCLUSIONS

Technical demands increase when reducing ApP, while locomotor demands increase when enlarging ApP. An ApP ~243 m^2^ · player is required to replicate the official match technical demands. Such an ApP was quite similar to the ApP required to replicate the official match high-speed locomotor demands. These findings might reinforce the knowledge about the use of specific ApP for soccer-specific training prescriptions using SSGs, and they might be useful to replicate, overload and underload both technical and locomotor demands using SSGs across the training routine.

### Practical applications

An ApP of ~243 m^2^ · player to ~288 m^2^ · player could be utilized to replicate concurrently official match demands for both technical and locomotor purposes. A different number of players (e.g. 5 vs 5, 7 vs 7, 8 vs 8) within the same ApP (i.e. ~243 to ~288 m^2^ · player) could be used to match both technical and physical demands accordingly with the technical-tactical aims of each drill. As a mere example, when training sessions aim to develop individual and team performance abilities (e.g., on match day -3 and/or -4), the use of a specific ApP may help to condition the players for match requirements towards different tactical learning environments.

Moreover, the present findings may also prove useful in congested-fixture periods [17], when time for training is lacking [41] and ball drills might be a viable conditioning option. For example, bouts of SSGs in specific large ApP should lead to higher external load demands than SSGs in a small ApP of similar time windows. This could enable an increase in either training volume or intensity, replicating the technical and locomotor official match demands in accordance with the distribution of official match intensities [42] without changes in the whole training session duration. Conversely, ApP < 243 m^2^ · player may help to overload the technical actions, reducing the locomotor demands when conditioning purposes are not enhanced. Therefore, since ApP ~243 m^2^ · player seems to be a good compromise between technical and locomotor activities to recreate the official match demands, practitioners could consistently utilize this specific ApP to recreate a highly contextualized training prescription based on the real-game model.
